# Impairment mechanism of nasal mucosa after radiotherapy for nasopharyngeal carcinoma

**DOI:** 10.3389/fonc.2022.1010131

**Published:** 2022-12-15

**Authors:** Caishan Fang, Yu Zhong, Tengyu Chen, Dan Li, Chunqiao Li, Xiangjun Qi, Junxia Zhu, Ruizhi Wang, Jinxiang Zhu, Shunlan Wang, Yan Ruan, Min Zhou

**Affiliations:** ^1^ The First Clinical Medical College, Guangzhou University of Chinese Medicine, Guangzhou, China; ^2^ Department of Radiotherapy, The First Affiliated Hospital of Guangzhou University of Chinese Medicine, Guangzhou, China; ^3^ Department of Otolaryngology, The Fifth Affiliated Hospital of Sun Yat-sen University, Zhuhai, China; ^4^ Department of Otolaryngology, The First Affiliated Hospital of Guangzhou University of Chinese Medicine, Guangzhou, China

**Keywords:** nasopharyngeal carcinoma, radiotherapy, nasal mucosa, epithelial barrier, impairment mechanism

## Abstract

The nasal mucosa, which performs the crucial functions of filtering, humidifying and temperature regulation, is one of the most vulnerable areas of nasopharyngeal carcinoma (NPC) patients after radiotherapy (RT). Following RT, NPC patients experience a series of pathological changes in the nasal mucosa, ultimately leading to physiological dysfunction of the nasal epithelium. This article systematically reviews the clinical and pathological manifestations of RT-related nasal damage in NPC patients and summarizes the potential mechanism of damage to the human nasal epithelium by RT. Finally, we outline the current mechanistic models of nasal epithelial alterations after RT in NPC patients and provide additional information to extend the in-depth study on the impairment mechanisms of the nasal mucosa resulting from RT. We also describe the relationship between structural and functional alterations in the nasal mucosa after RT to help mitigate and treat this damage and provide insights informing future clinical and fundamental investigations.

## 1 Introduction

Nasopharyngeal carcinoma (NPC) is an epithelial carcinoma originating from the nasopharyngeal epithelium (1). NPC has extremely high incidence rates in Southeast Asia and North Africa, with over 70% of new cases occurring in these regions, especially in China, in 47.7% of cases occur (2–4). The occurrence of NPC has been proven to be related to genetic, ethnic and environmental factors and Epstein-Barr virus (EBV) infection. There is a higher incidence of NPC in males than females, with a ratio of 2:5 in China in 2015 (5, 6). NPC is very sensitive to radiotherapy (RT), unlike other head and neck malignancies. Therefore, the most classic treatment for NPC is RT in both the early and advanced stages of the disease (7, 8). Early conventional RT techniques cause severe side effects, including mucositis, dermatitis, xerostomia and dysphagia (4, 9). RT is also associated with late toxic effects, including xerostomia, sensorineural hearing loss, osteoradionecrosis, trismus, Central nervous system (CNS) abnormalities and hormonal dysfunction (4, 10). As the radiation dose increases or accumulates, the toxic side effects become more obvious (10). However, photon-based RT techniques have evolved from traditional two-dimensional RT to three-dimensional conformal RT to intensity-modulated RT (IMRT). Although RT techniques have advanced greatly and the precise radiation range of IMRT causes less damage to normal tissues, toxic side effects are still common in surviving patients.

As one of the most vulnerable areas to IMRT or common RT, the nasal mucosa is substantially damaged upon RT initiation. When NPC patients receive RT, the tumour cells are killed, but direct damage is also sustained in tumour-adjacent areas such as the nasal mucosa (11). RT usually causes congestion and oedema of the nasal mucous membrane, discharge of purulent secretions from the nasal cavity, adhesion of mucus in the nasal cavity and sinus orifices, and damage and loss of cilia. Then, a series of inflammatory reactions result in sinus drainage obstruction, eventually leading to nasal diseases such as radiation-induced chronic rhinosinusitis (CRSr) (12). These patients mainly present with symptoms similar to chronic rhinosinusitis (CRS), such as nasal congestion, rhinorrhoea, smell loss, headache, and head swelling (10). Acute onset may be accompanied by additional symptoms such as fever and rhinorrhoea (10, 13). The incidence of CRSr was as high as 86.1% with past techniques (14). Despite the improvement of RT techniques in recent years, the incidence of CRSr caused by moderate-intensity RT is still as high as 73.5%, and the damage has a cumulative effect, which usually reaches a maximum after 1 year (12, 15). Mechanistically, the consensus view attributes CRSr to radiation damage sustained by the ciliated cells and goblet cells of the nasal mucosa (16). Many studies have focused on the damage to the nasal mucosa caused by RT, but there is still a lack of high-quality evidence to confirm the association between clinical manifestations and pathological changes after RT. This review is a comprehensive overview of the important findings regarding damage to the nasal mucosa after RT and systematically organizes and categorizes the clinical and pathological manifestations of nasal diseases after RT.

## 2 Clinical characteristics of NPC patients after treatment with RT

**Figure 1:**
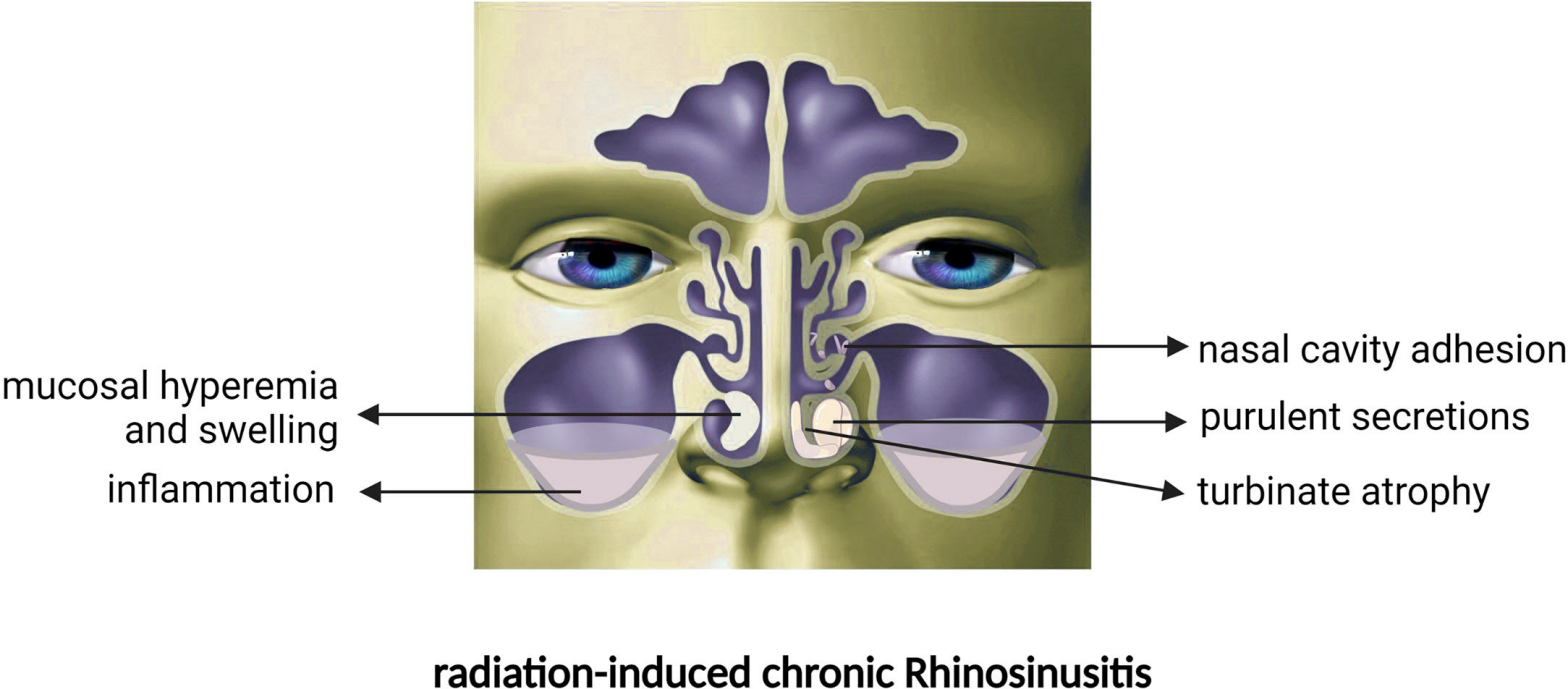
Nasal manifestations after RT in NPC patients.

After RT, purulent nasal discharge, nasal congestion, and significant anosmia (16, 17) ultimately lead to nasal disease and low quality of life (9, 18, 19) in NPC patients (**Figure 1**). Numerous studies (20–22) have verified a significant decrease in nasal ciliary clearance after RT through the saccharin/charcoal test. Ciliary clearance dysfunction causes NPC patients to develop nasal congestion, facial pain and the perception of unpleasant odour; furthermore, the prolonged clearance time indicates cilia dysfunction or reduction in number, which leads to inadequate drainage of secretions. Moreover, NPC patients are vulnerable to upper respiratory tract infections after RT due to the impaired nasal epithelial barrier (17, 23).

RT also has other serious side effects in the early stage, such as acute reactions in the oral mucosa, which lead to difficulty swallowing and eating and pain in the throat. Hearing loss, dry mouth, nasal dryness and hypothyroidism also occur in the early stage of RT (9, 19, 24, 25). Notably, physical problems may not be the only symptoms occurring after RT. Even after the physical complications subside, survivors still face many psychosocial challenges, such as fatigue, cognitive changes, emotional distress, occupational difficulties, sexual dysfunction and fear of cancer recurrence (9, 10, 26).

Among nasal problems after RT, hyposmia is a notable complication relevant to nasal mucosal injury. Giuseppe Riva et al. (27)discovered that because of acute inflammation of the nasal mucosa, patients developed nasal congestion, and their sense of smell was correspondingly diminished. Radiation causes patients’ nasal mucosa to undergo various changes, such as congestion, oedema and mucosal thickening. These changes prevent odours from entering the olfactory area and lead to conductive hyposmia in the early stage of RT (13, 27). Olfactory dysfunction (OD) is classified into transport, sensory, and neural disorders (28). The mechanism of olfactory disorders after RT (17, 27) may include both transport and sensory disorders. When RT is completed, the acute inflammation of the patient’s nasal mucosa will subside, and the olfactory disturbance caused by transport disruption will recover (28).

Therefore, in the early radiation stage, transport disorders can be accounted for the mechanical obstruction of odorants to the olfactory cleft caused by edema. With the gradual accumulation of radioactive Impairment, we can speculate that the nasal mucosa is irreversibly damaged by the stimulation of Long-term chronic inflammation. Due to abnormal repair of the nasal epithelium, the squamous epithelium replaces the normal tissue. This phenomenon impairs olfactory neurosensory function, and this impairment progresses to complete loss of smell. However, this is merely our speculation based on previous studies; future research should test this hypothesis further.

## 3 Endoscopic manifestation and nasal imaging changes after RT

### 3.1 Nasal endoscopic manifestations

**Figure 2:**
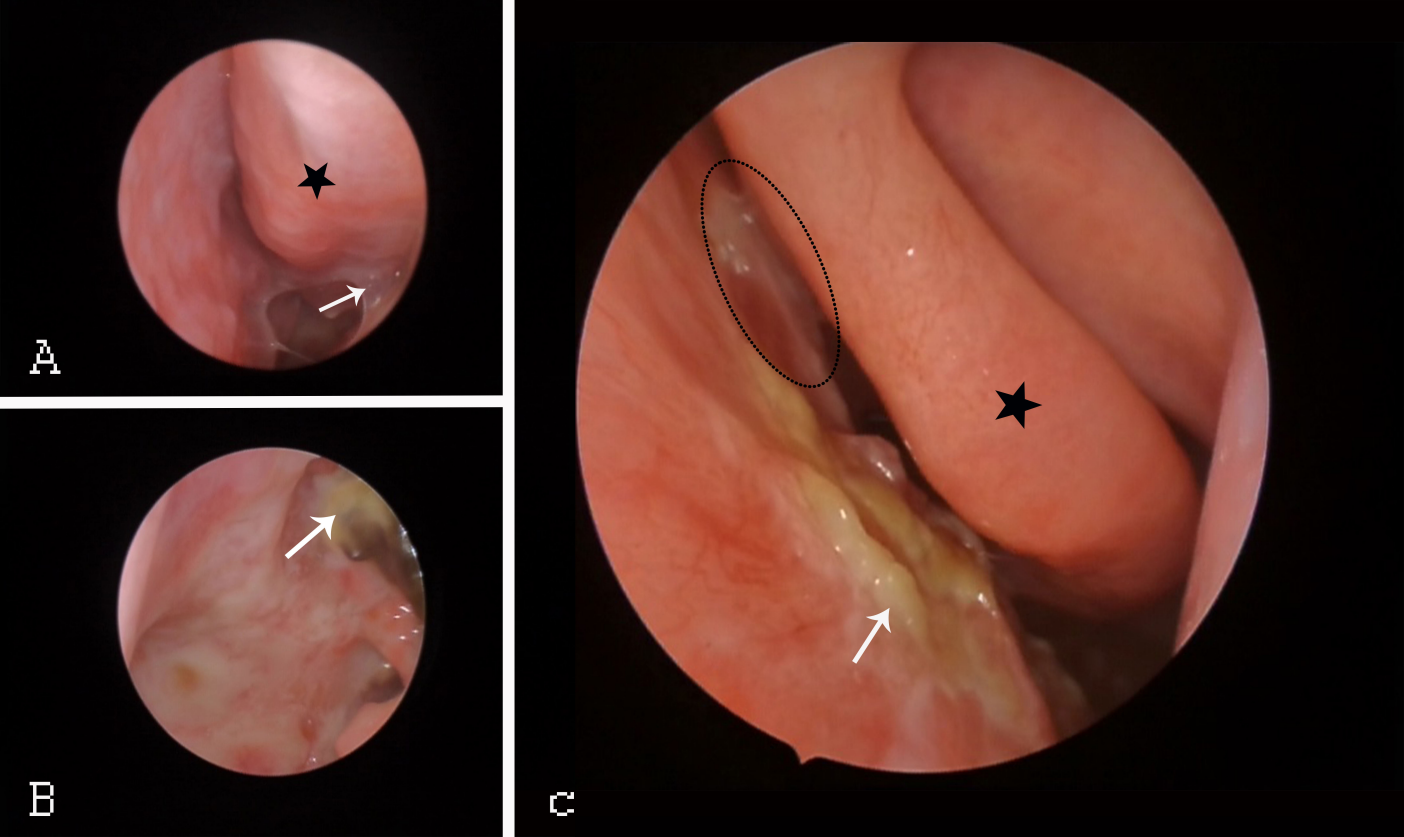
Endoscopic changes in NPC patients after RT. **(A)** Black star: congested IT. White arrow: adhesions throughout the nasal tract. **(B)** White arrow: pharyngeal fossa in the nasopharynx. **(C)** Black asterisk: MT. White arrow: middle nasal tract with purulent secretions and scabs. Black ellipse: middle nasal tract with nasal adhesions. IT, inferior turbinate; MT, middle turbinate.

Regular nasal endoscopy facilitates visual monitoring of NPC recurrence and reveals continuous changes in the nasal mucosa after RT. Endoscopy(**Figure 2**) can identify the occurrence of sinusitis so that treatment can be provided promptly. Reda Kamel et al. (29)found delayed damage to the nasal mucosa within 2-6 weeks after patients completed RT. The nasal mucosa was continuously congested and oedematous, and after some time, it began to show the development of more purulent secretions, scabs, and adhesions; atrophy of the turbinates, widening of the sinus openings (especially the maxillary sinus and anterior ethmoid sinus) and narrowing or atresia of the posterior nostrils.

### 3.2 CT and MRI representation of the nasal mucosa after RT

Computed tomography (CT) imaging is a crucial tool for diagnosing rhinosinusitis; this technique allows clinicians to observe the severity of a patient’s sinus mucosal inflammation (25, 30). Sinusitis is a common side effect after RT in patients with nasopharyngeal cancer. When patients develop nasal symptoms such as nasal congestion and rhinorrhoea after RT, CT imaging of the paranasal sinuses can be reviewed to assess the inflammation of the nasal mucosa and associated sinuses. In a report by Reda Kamel et al. (29)describing 32 NPC patients who received RT and had no recurrence, CT results also showed that inflammation of the sinus mucosa was present in most patients after RT. The highest incidence of inflammation was in the maxillary sinuses, followed by the anterior septum, the ostiomeatal complex (OMC), and the posterior septum, with the lowest incidence in the frontal sinuses. CT showed that the maxillary sinuses, anterior septal sinus and OMC were the most affected areas (29, 31).

**Figure 3:**
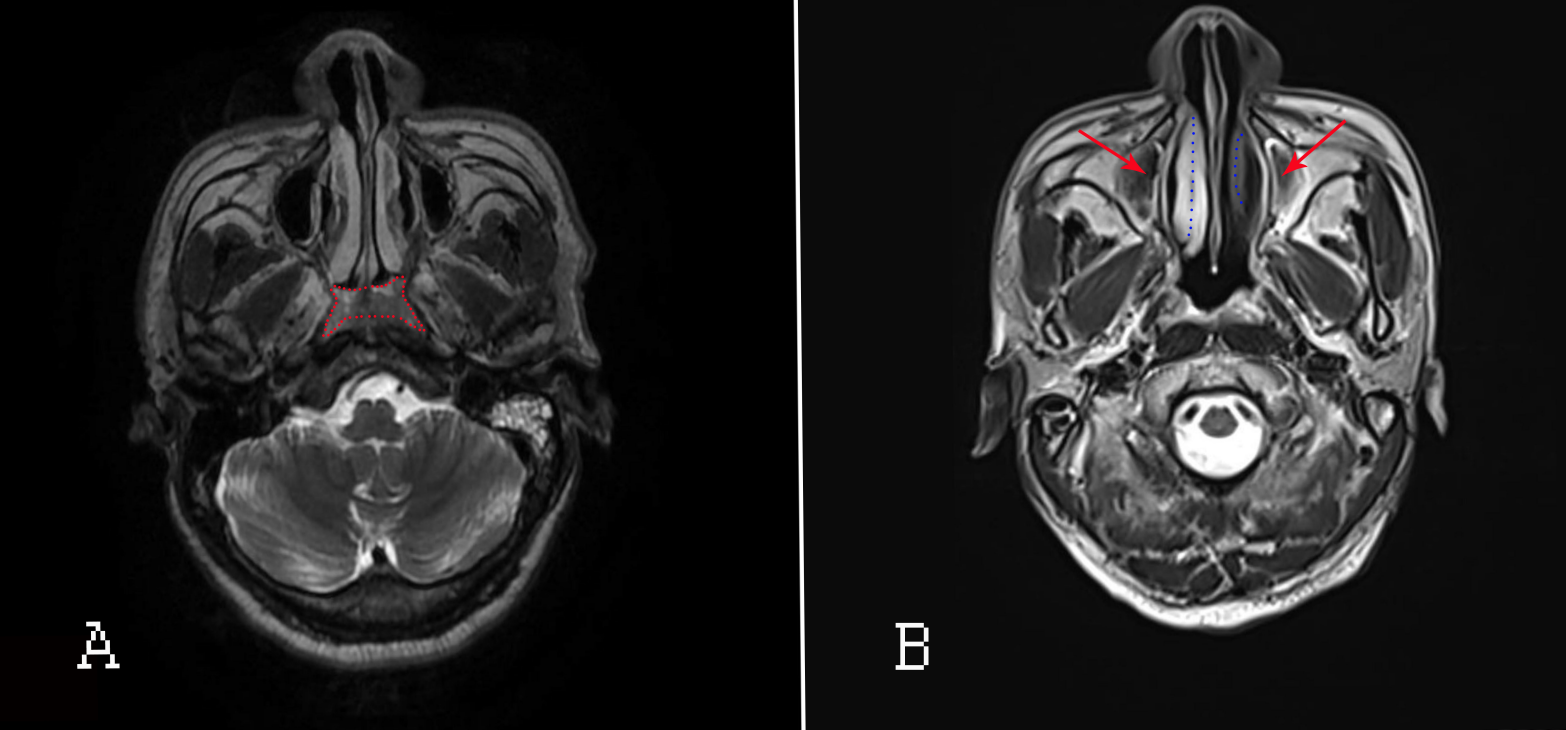
MRI of the nasopharyngeal passages and skull base. **(A)** Before RT; the red area indicates nasopharyngeal lesions. **(B)** After RT. Red arrows: inflammation of the maxillary sinus; blue dotted lines: oedematous IT (right) and atrophied IT (left).

Accurate delineation of tumor extent is a key step in determining NPC staging and treatment strategy (32, 33). Certainly, in this field, head and neck magnetic resonance imaging (HN-MRI) has been regarded as the best modality for assessing local NPC extension. Its influence on the tumor (T) and nodal (N) staging system of NPC is more pronounced than that of CT (32, 33). In addition, HN-MRI examinations should be performed regularly after RT for NPC patients. The MRI(**Figure 3**) manifestation in NPC patients with paranasal sinus invasion include damage to sinus boundary, uneven thickening of the sinus mucosa or with massive effusion in the sinus cavity (34). MRI is highly recommended for observing the size of local nasopharyngeal swelling lesions and the scope and degree of invasion of surrounding adjacent tissues. MRI also improves the accuracy of diagnosis of NPC (35–37). However, for detecting extent of nasal inflammatory response and lesions after RT, HN-MRI is recognized not as well visualized as CT (since CT is reported as the gold standard modality for CRS) in observing the construction of nasal cavity and/or paranasal sinuses (35, 38).

## 4 Pathological changes in the nasal mucosa after RT for NPC

### 4.1 Morphological structure and function of the normal nasal mucosa

The normal nasal mucosa has a certain normal histological pattern. After conventional haematoxylin-eosin staining and periodic acid–Schiff (PAS) staining, normal nasal mucosal tissue can be observed to contain ciliated cells in the epithelial layer, while the glands and glandular cells are in the basal and lamina propria layers (16, 39). The epithelial layer is dominated by neatly arranged and dense ciliated columnar cells. The cilia are densely arranged, neat and uniformly oriented. Small concentrated areas of goblet cells and scattered single goblet cells are seen between the columns. Basal cells are neatly arranged without obvious hyperplasia.

In the intrinsic layer, there are mixed, nonatrophied serous and mucus glands, with serous glands being more common and occurring mainly in the lobules, while mucus glands are less common and occur in the submucosa and deeper interstitium. The interstitium is loose connective tissue, denser in the deep part than in the superficial part, with a few collagen fibres in the deep part and awn-like interstitial cells in the superficial part. More capillaries, nerve endings, a few lymphocytes and plasma cells can be seen in the intrinsic layer. Eosinophils and granulocytes are occasionally seen as well. Electron microscopy shows that the cilia have a neat, dense, uniform, dense and neat distribution and a consistent direction. On transmission electron microscopy, most nasal epithelial layers are closely arranged with ciliated columnar epithelial cells with a normal nucleoplasmic ratio; the nuclei are normal in size and morphology, containing mainly euchromatin; an abundant rough endoplasmic reticulum and numerous mitochondria are seen in the cytoplasm (16, 40).

The main functions of the structurally normal nose are respiration, heating and moistening of inhaled air, olfaction, vocalization and mucociliary clearance (MCC). If the nasal mucosal blood supply, nerves, glands and mucociliary system function normally, air is inhaled through the nose during respiration and then maintains sufficient pressure, volume, humidity, heat and cleanliness as it travels to the lungs (22, 41).

### 4.2 Variation in the epithelial structure of the nasal mucosa after RT for NPC

#### 4.2.1 Alterations in ciliary cells

Cilia are important constituent structures of the upper respiratory tract, especially in the nose. They fulfil the physiological function of clearing out foreign bodies and microbes. When the morphology and function of cilia are impaired, it can cause a series of pathological phenomena, such as bronchial dilatation, sinusitis, and nasal polyps. When NPC patients receive RT, radiation can directly damage ciliated cells and cause a significant decrease in the quantity and function of ciliated cells. Many scholars (17, 20, 21, 23, 29, 42) have applied the saccharin test or a modified version of the saccharin test, and the results showed prolonged transport time of cilia and reduced mucociliary clearance, indicating that RT could damage ciliated cells. Surico G et al. (23) confirmed that this damage was permanent. PenJen Lou (43)and Suizi Zhou (16) et al. observed that the architecture of cilia was disorganized, sparse, absent or diverging after RT *via* light microscopy (LM) and transmission electron microscopy (TEM). Moreover, impaired ciliary motility was also observed in the histological examination of nasal biopsy tissue (11, 16). Lou, PJ et al. (43) found delayed impairment effects of radiation in the nasal mucosa after RT. Previous results showed increased deposition of dense collagen fibres in the lamina propria under light microscopy after RT, along with a stratified arrangement of epithelial cells and a gradual decrease in cytoplasmic volume. Ultrastructural observations could detect cilia area loss, inter-and intracellular vacuole formation and ciliary aberrations. Researchers have also observed these pathological phenomena in NPC patients after 23 years of RT, suggesting that this radiation-induced damage to the nasal epithelium was irreversible and had a cumulative effect with time (43). Damage to the nasal mucosa can further lead to rhinosinusitis, which is why the majority of patients had the highest incidence of sinusitis 1 year after the end of RT (7, 12, 44).

There are a set of markers for ciliated cells, such as alpha-tubulin, beta-tubulin and TAp73 (45–47), and markers of microtubule assemblies of motile cilia, such as DNAH5, DNAI1 and RSPH4A (48–50). Ciliogenesis-associated molecular markers include FOXJ1 and CP110 (51, 52). According to previous research, DNAH5 or DNAI1 mutations contribute to outer dynein arm defects (53–55), while RSPH4A encodes protein components of the axonemal radial spoke on the head of the cilium (49). Foxj1 and Cp110 are responsible for abnormal ciliogenesis (53, 56). In recent years, the mechanism by which RT damages the nasal mucosa has been investigated using these biomarkers, first qualitatively and then quantitatively. Hongming Huang (42) and Suizi Zhou et al. (16)detected these biomarkers in nasal epithelial cells after RT. Their results showed that alpha-tubulin, beta-tubulin, TAp73, DNAH5, DNAI1, RSPH4A, FOXJ1 and CP110 levels were significantly reduced. This result indicated that RT-induced impairment was ascribable to the reduced production of ciliated cells or that the generation of ciliated cells was damaged. In addition, these remaining ciliated cells lose their oscillatory function due to the inactivation of ciliated power arms, resulting in diminished mucociliary clearance. More in-depth studies are needed to elucidate the mechanism of the decreased ciliated cells, prolonged transport time and diminished mucociliary clearance.

#### 4.2.2 Alterations in goblet cells

Goblet cells are mucus-secreting cells that synthesize and secrete mucins to form a mucosal barrier that protects nasal epithelial cells. Radiation can likewise damage goblet cells and affect their secretory function. The quantity of normal goblet cells is also significantly reduced after RT, and the mucous glands tend to atrophy, which is compensated by dysplastic goblet cells, according to observations under light microscopy (16, 42). On TEM, normal goblet cells appear vacuolated, degenerated and even atrophied. The common biomarkers of goblet cells are MUC5AC and MUC5B (16, 42, 53, 57). Hongming Huang (42) and Suizi Zhou et al (16) collected nasal mucosal tissues of NPC patients after RT for haematoxylin and eosin (HE), immunohistochemistry and fluorescence staining. They found that MUC5AC and MUC5B levels in nasal mucosal tissues of NPC patients were also significantly reduced. Ko-Hsin Hu et al (58) showed that the submucosal gland openings decreased after RT.

#### 4.2.3 Alterations in basal cells

**Figure 4:**
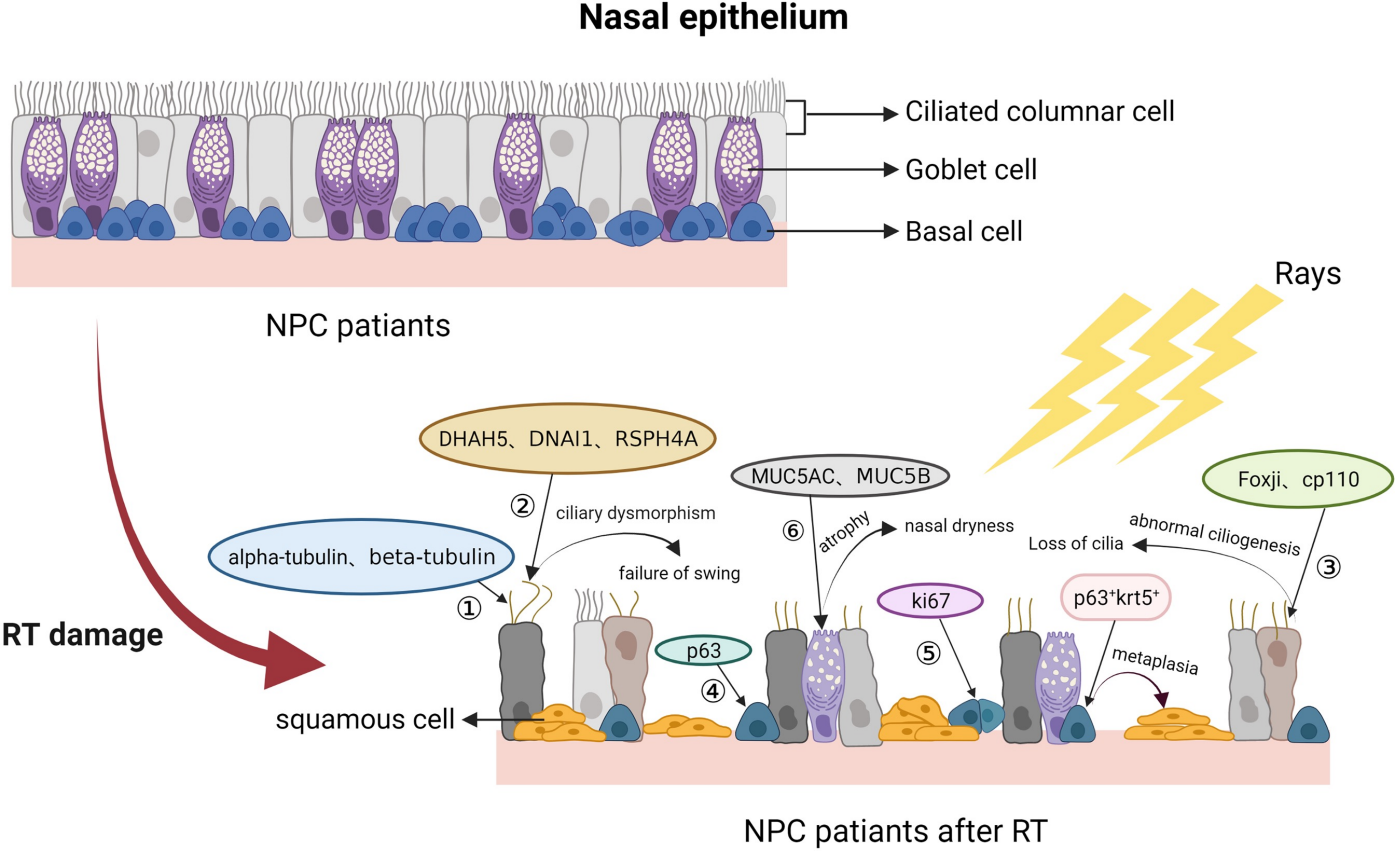
NPC patients after RT. After RT, nasal ciliated columnar cells of NPC patients gradually decrease in number and lose ciliary motility, goblet cells decrease in number or abnormally proliferate, and basal cells decrease in number or give way to squamous metaplasia. ① Biomarkers of ciliated columnar cells; ② Biomarkers of basal microtubule assemblies; ③ Biomarkers associated with ciliogenesis; ④ Biomarkers of basal cells; ⑤ Biomarkers of cell proliferation.

Basal cells, as nasal stem/progenitor cells, have the function of differentiating into other types of functional nasal epithelial cells. In general, the nasal mucosa is constantly renewed and maintains self-repair capacity, ascribed to basal cell proliferation and differentiation. Reduced basal cell numbers and widening of the intercellular space between basal cells in NPC patients after RT have also been observed by light microscopy and TEM. The biomarkers of basal cells are P63 and Krt5 (42, 59). Ki67 can also be a biomarker of nasal basal cells in the proliferative phase (16, 53). A related study showed an increase in the numbers of P63+/Krt5+ cells and a decrease in the numbers of Ki67 cells in the nasal mucosa after RT (42). In summary, in addition to impairing ciliated and goblet cells, RT can also reduce the proliferative capacity of basal cells and lead to their abnormal proliferation. Due to genetic mutations, it is difficult for damaged basal cells to differentiate into normal ciliated cells and goblet cells to repair the defective nasal mucosal epithelium; this deficiency eventually promotes squamous metaplasia of nasal cells, leading to structural remodelling of the nasal epithelium. Pathological changes of nasal mucosa after radiotherapy in NPC patients are showed in **Figure 4**.

#### 4.2.4 Other microlevel effect

In addition to changes in the structure of nasal epithelium, NPC patients have other microlevel performance when suffering from CRSr. Kuhar (11) et al. reported no difference in eosinophil counts or neutrophilic infiltration between CRSr patients and CRS patients without nasal polyps (CRSsNP patients). when comparing with CRS patients with nasal polyps (CRSwNP patients), CRSr patients exhibited decreased eosinophilia and eosinophil aggregates. There was no difference in the overall degree of inflammation and fibrosis among three groups. It may indicate that there is distinct underlying mechanisms and histopathologic changes in CRSr, and its endotypes is diffirent from CRSsNP or CRSwNP. However, Giuseppe Riva (27) et al. performed nasal cytology, which showed prominent neutrophils and bacteria in the nasal mucosa after CRSr. Stoddard (60) et al. researched the microbial flora of CRSr patients using cell culture and molecular techniques for microbial DNA detection. The bacteria in CRSr patients resembled the microorganisms responsible for common CRS. The most common organism identified was *Staphylococcus aureus*, followed by *Pseudomonas aeruginosa*. The reasons for the discrepancy in these researches can be explained that there is a lack of studies related to the endotype or molecular immune response to CRSr. Thus, follow-up work with greater sample sizes and larger power are needed to confirm them.

The toxic effects of radiation on tumor cells also change the tumor microenvironment. T cells, B cells and NK cells is very radiosensitive after RT. It helps the recruitment of antitumor T cells to against tumor (61). Meanwhile, RT has the potential to trigger type I interferon response and DNA damage which also help rebuild the antitumor immune defence (62). The immune disfunction caused by radiation also results in a maladjusted immune system and unbalanced growth of parasitic bacteria in the human body. Several harmful bacteria can propagate rapidly. Due to the proximity of the nasal cavity to the nasopharynx, the intensity of radiation exposure in the nasal cavity is relatively high. Nasal epithelia are prone to damage from immune disorders, and microbial dysbiosis occurs due to radiation damage (63, 64). However, the interacting genes and pathways involved in radiation impairment associated with the nasal epithelial barrier and the immune system leading to rhinosinusitis need to be addressed in future research.

## 5 Impairment mechanism of nasal mucosa after RT for NPC

Therefore, based on previous study, it suggests that the occurrence of radioactive sinusitis has a close relationship with the impairment to nasal epithelial barrier. It exists two possible causes of radiation damage to the nasal epithelial barrier. One reason is the direct killing effect of radiation on the epithelial cells which leads to the depletion of cilia, goblet cells and basal cells (16, 63). Another cause may be the contribution of radiation to acute inflammation (61, 64). The release and stimulation of plenty of inflammatory factors gradually lead to atrophy and death of epithelia. The defective and damaged nasal epithelial barrier further leads to epithelial dysfunction, resulting in rhinosinusitis.

## 6 Conclusions

After NPC patients undergo RT, the clinical characteristics and microenvironment of the nasal mucosa, including morphological structure and function, has been altered obviously. As the damage to the nasal epithelial barrier accumulates and immune function declines, CRSr occurs. It has been emphasized the relationship between structural alterations and functional impairment of the nasal mucosa after RT in this article. However, the mechanism for RT promoting the damage and loss of nasal functional epithelial cells (ciliary cells, goblet cells and basal cells) still remains unclear. Moreover, the interrelationship between the loss of ciliated columnar epithelium, the decrease in goblet cells, the genetic changes in basal cells and squamous metaplasia has not yet been confirmed.

Consequently, how to effectively prevent and treat CRSr remains a substantial pressing challenge in clinical practice. Understanding the changes in local microstructures, the microenvironment and systemic immune responses is of great significance in dealing with nasal mucosal injuries after RT. Meanwhile, RT-induced impairment to nasal microstructures in NPC patients is a potential entry point for in-depth research, and further exploration of the mechanism of nasal mucosal injury by RT or IMRT needs to be addressed.

## Author contributions

CF, YZ, and TC developed the idea, collected the data, and constructed graphs; CF wrote the manuscript. CL, XQ and DL collected the data and wrote the manuscript. DL, JuZ, RW, and JiZ revised the manuscript. SW and YR contributed to funding acquisition. MZ was responsible for supervision. All authors have read and approved the final manuscript.

## References

[1] Chua M Wee J Hui EP Chan A . Nasopharyngeal carcinoma. LANCET (2016) 387:1012–24. doi: 10.1016/S0140-6736(15)00055-0 26321262

[2] Wong K Hui EP Lo KW Lam WKJ Johnson D Li L . Nasopharyngeal carcinoma: an evolving paradigm. Nat Rev Clin Oncol (2021) 18:679–95. doi: 10.1038/s41571-021-00524-x 34194007

[3] Bray F Ferlay J Soerjomataram I Siegel RL Torre LA Jemal A . Global cancer statistics 2018: GLOBOCAN estimates of incidence and mortality worldwide for 36 cancers in 185 countries. CA Cancer J Clin (2018) 68:394–424. doi: 10.3322/caac.21492 30207593

[4] Chen YP Chan A Le QT Blanchard P Sun Y Ma J . Nasopharyngeal carcinoma. LANCET (2019) 394:64–80. doi: 10.1016/S0140-6736(19)30956-0 31178151

[5] Chen W Zheng R Baade PD Zhang S Zeng H Bray F . Cancer statistics in China, 2015. CA Cancer J Clin (2016) 66:115–32. doi: 10.3322/caac.21338 26808342

[6] Zhang G Zong J Lin S Verhoeven RJ Tong S Chen Y . Circulating Epstein-Barr virus microRNAs miR-BART7 and miR-BART13 as biomarkers for nasopharyngeal carcinoma diagnosis and treatment. Int J Cancer (2015) 136:E301–12. doi: 10.1002/ijc.29206 25213622

[7] Huang C Huang M Shih MP Cheng KY Lee KW Lu TY . Post-radiation sinusitis is associated with recurrence in nasopharyngeal carcinoma patients treated with intensity-modulated radiation therapy. Radiat Oncol (2019) 14(8). doi: 10.1186/s13014-019-1261-9 PMC6458621 30971260

[8] Siegel RL Miller KD Fuchs HE Jemal A . Cancer statistics, 2021. CA Cancer J Clin (2021) 71:7–33. doi: 10.3322/caac.21654 33433946

[9] McDowell L Corry J Ringash J Rischin D . Quality of life, toxicity and unmet needs in nasopharyngeal cancer survivors. Front Oncol (2020) 10:930. doi: 10.3389/fonc.2020.00930 32596155 PMC7303258

[10] Tuan JK Ha TC Ong WS Siow TR Tham IW Yap SP . Late toxicities after conventional radiation therapy alone for nasopharyngeal carcinoma. RADIOTHER Oncol (2012) 104:305–11. doi: 10.1016/j.radonc.2011.12.028 22280806

[11] Kuhar HN Tajudeen BA Heilingoetter A Mahdavinia M Gattuso P Ghai R . Distinct histopathologic features of radiation-induced chronic sinusitis. Int Forum Allergy RH (2017) 7:990–8. doi: 10.1002/alr.21989 28736997

[12] Su YX Liu LP Li L Li X Cao XJ Dong W . Factors influencing the incidence of sinusitis in nasopharyngeal carcinoma patients after intensity-modulated radiation therapy. Eur Arch Otorhinolaryngol (2014) 271:3195–201. doi: 10.1007/s00405-014-3004-8 24659365

[13] Riva G Boita M Ravera M Moretto F Badellino S Rampino M . Nasal cytological changes as late effects of radiotherapy for nasopharyngeal cancer. Am J Rhinol Allergy (2015) 29:e41–5. doi: 10.2500/ajra.2015.29.4156 25785741

[14] Luo HH Fu ZC Cheng HH Liao SG Li DS Cheng LP . Clinical observation and quality of life in terms of nasal sinusitis after radiotherapy for nasopharyngeal carcinoma: long-term results from different nasal irrigation techniques. Br J Radiol (2014) 87:20140043. doi: 10.1259/bjr.20140043 24814695 PMC4075587

[15] Ayoub N Walgama E Thamboo A Nayak JV Hwang PH . Efficacy of endoscopic sinus surgery for chronic rhinosinusitis following primary radiotherapy and concurrent chemotherapy for nasopharyngeal carcinoma. Int Forum Allergy Rhinol (2017) 7:1045–51. doi: 10.1002/alr.22002 28806502

[16] Zhou S Huang H Chen Q Tan KS Zhu Z Peng Y . Long-term defects of nasal epithelium barrier functions in patients with nasopharyngeal carcinoma post chemo-radiotherapy. RADIOTHER Oncol (2020) 148:116–25. doi: 10.1016/j.radonc.2020.03.038 32353641

[17] Gurushekar PR Isiah R John S Sebastian T Varghese L . Effects of radiotherapy on olfaction and nasal function in head and neck cancer patients. Am J OTOLARYNG (2020) 41:102537. doi: 10.1016/j.amjoto.2020.102537 32416968

[18] Hong JS Tian J Han QF Ni QY . Quality of life of nasopharyngeal cancer survivors in China. Curr Oncol (2015) 22:e142–7. doi: 10.3747/co.22.2323 PMC4462535 26089724

[19] Tyler MA Mohamed A Smith JB Aymard JM Fuller CD Phan J . Long-term quality of life after definitive treatment of sinonasal and nasopharyngeal malignancies. LARYNGOSCOPE (2020) 130:86–93. doi: 10.1002/lary.27849 30706478

[20] Yin G Tu B Ye L . Correlation of intensity-modulated radiation therapy at a specific radiation dose with the prognosis of nasal mucous damage after radiotherapy. Radiat Environ Biophys (2020) 59:245–55. doi: 10.1007/s00411-020-00830-5 32030481

[21] Johnston M Yu E Kim J . Perineural invasion and spread in head and neck cancer. Expert Rev ANTICANC (2014) 12:359–71. doi: 10.1586/era.12.9 22369327

[22] Kılıç C Tunçel ÜVerifytat Cömert E Kaya BV . The effect of radiotherapy on mucociliary clearance in patients with laryngeal and nasopharyngeal cancer. Eur Arch OTO-RHINO-L (2015) 272:1517–20. doi: 10.1007/s00405-014-3082-7 24838358

[23] Surico G Muggeo P Mappa L Muggeo V Conti V Lucarelli A . Impairment of nasal mucociliary clearance after radiotherapy for childhood head cancer. Head Neck (2001) 23:461–6. doi: 10.1002/hed.1060 11360307

[24] Huang TL Tsai MH Chuang HC Chien CY Lin YT Tsai WL . Quality of life and survival outcome for patients with nasopharyngeal carcinoma treated by volumetric-modulated arc therapy versus intensity-modulated radiotherapy. Radiat Oncol (2020) 15:84. doi: 10.1186/s13014-020-01532-4 32307024 PMC7168825

[25] Lim S Ramirez MV Garneau JC Ford MK McKeough K Ginat DT . Three-dimensional image analysis for staging chronic rhinosinusitis. Int Forum Allergy Rhinol (2017) 7:1052–7. doi: 10.1002/alr.22014 PMC5965263 28941169

[26] Nguyen NA Ringash J . Head and neck cancer survivorship care: A review of the current guidelines and remaining unmet needs. Curr Treat Options Oncol (2018) 19:44. doi: 10.1007/s11864-018-0554-9 29987676

[27] Riva G Franco P Provenzano E Arcadipane F Bartoli C Lava P . Radiation-induced rhinitis: Cytological and olfactory changes. Am J Rhinol Allergy (2019) 33:153–61. doi: 10.1177/1945892418822448 30632393

[28] Whitcroft KL Hummel T . Clinical diagnosis and current management strategies for olfactory dysfunction: A review. JAMA Otolaryngol Head Neck Surg (2019) 145:846–53. doi: 10.1001/jamaoto.2019.1728 31318413

[29] Kamel R Al-Badawy S Khairy A Kandil T Sabry A . Nasal and paranasal sinus changes after radiotherapy for nasopharyngeal carcinoma. Acta OTO-LARYNGOL (2004) 124:532–5. doi: 10.1080/00016480410018106 15224889

[30] Greguric T Prokopakis E Vlastos I Doulaptsi M Cingi C Košec A . Imaging in chronic rhinosinusitis: A systematic review of MRI and CT diagnostic accuracy and reliability in severity staging. J Neuroradiol (2021) 48:277–81. doi: 10.1016/j.neurad.2021.01.010 33539844

[31] Wu PW Huang CC Lee YS Chou YC Fan KH Lin CY . Post-irradiation sinus mucosa disease in nasopharyngeal carcinoma patients treated with intensity-modulated proton therapy. Cancers (Basel) (2022) 14(225):1–3. doi: 10.3390/cancers14010225 PMC8750360 35008389

[32] Gihbid A Cherkaoui Salhi G El Alami I Belgadir H Tawfiq N Bendahou K . Pretreatment [18F]FDG PET/CT and MRI in the prognosis of nasopharyngeal carcinoma. Ann Nucl Med (2022) 36:876–886. doi: 10.1007/s12149-022-01770-4 35836088

[33] Tang LL Chen YP Chen CB Chen MY Chen NY Chen XZ . The Chinese society of clinical oncology (CSCO) clinical guidelines for the diagnosis and treatment of nasopharyngeal carcinoma. Cancer Commun (Lond) (2021) 41:1195–227. doi: 10.1002/cac2.12218 PMC8626602 34699681

[34] Tian L Mo YX Li YZ Liu LZ Fan W . [MRI analysis of paranasal sinus invasion in 182 patients with nasopharyngeal carcinoma]. Zhonghua Yi Xue Za Zhi (2013) 93:3779–82. doi: 10.3760/cma.j.issn 24548398

[35] Abdel KARA King A . MRI And CT of nasopharyngeal carcinoma. AJR Am J Roentgenol (2012) 198:11–8. doi: 10.2214/AJR.11.6954 22194474

[36] Chan SC Yeh CH Yen TC Ng SH Chang JT Lin CY . Clinical utility of simultaneous whole-body (18)F-FDG PET/MRI as a single-step imaging modality in the staging of primary nasopharyngeal carcinoma. Eur J Nucl Med Mol Imaging (2018) 45:1297–308. doi: 10.1007/s00259-018-3986-3 29502310

[37] Chen WS Li JJ Hong L Xing ZB Wang F Li CQ . Comparison of MRI, CT and 18F-FDG PET/CT in the diagnosis of local and metastatic of nasopharyngeal carcinomas: an updated meta analysis of clinical studies. Am J Transl Res (2016) 8:4532–47. PMC5126302 27904660

[38] Chakraborty P Jain RK . Nasal endoscopy as an effective alternative for CT-scan in diagnosing chronic rhinosinusitis: A clinical study and review of literature. Indian J Otolaryngol Head Neck Surg (2019) 71:1734–8. doi: 10.1007/s12070-017-1085-6 PMC6848606 31763235

[39] Li J Lin X Liu X Ma Z Li Y . Baicalin regulates Treg/Th17 cell imbalance by inhibiting autophagy in allergic rhinitis. Mol Immunol (2020) 125:162–71. doi: 10.1016/j.molimm.2020.07.008 32688118

[40] Pagliuca G Rosato C Martellucci S de Vincentiis M Greco A Fusconi M . Cytologic and functional alterations of nasal mucosa in smokers: temporary or permanent damage? Otolaryngol Head Neck Surg (2015) 152:740–5. doi: 10.1177/0194599814566598 25573681

[41] Varricchio A Avvisati F Varricchio AM Tortoriello G Ciprandi G . The nose and paranasal sinuses. Int J Immunopathol Pharmacol (2010) 23:1–3. 20152069

[42] Huang H Tan KS Zhou S Yuan T Liu J Ong HH . p63+Krt5+ basal cells are increased in the squamous metaplastic epithelium of patients with radiation-induced chronic rhinosinusitis. Radiat Oncol (2020) 15. doi: 10.1186/s13014-020-01656-7 PMC7517817 32977822

[43] Lou PJ Chen WP Tai CC . Delayed irradiation effects on nasal epithelium in patients with nasopharyngeal carcinoma. an ultrastructural study. Ann Otol Rhinol Laryngol (1999) 108:474–80. doi: 10.1177/000348949910800510 10335709

[44] Huang CC Huang SF Lee TJ Ng SH Chang JT . Postirradiation sinus mucosa disease in nasopharyngeal carcinoma patients. LARYNGOSCOPE (2007) 117:737–42. doi: 10.1097/MLG.0b013e3180325b6c 17334261

[45] Maurya AK Rogers T Sengupta PA . CCRK and a MAK kinase modulate cilia branching and length *via* regulation of axonemal microtubule dynamics in caenorhabditis elegans. Curr Biol (2019) 29:1286–300. doi: 10.1016/j.cub.2019.02.062 PMC6482063 30955935

[46] Li CW Shi L Zhang KK Li TY Lin ZB Lim MK . Role of p63/p73 in epithelial remodeling and their response to steroid treatment in nasal polyposis. J Allergy Clin Immunol (2011) 127:765–72. doi: 10.1016/j.jaci.2010.12.011 21269671

[47] Raidt J Werner C Menchen T Dougherty GW Olbrich H Loges NT . Ciliary function and motor protein composition of human fallopian tubes. Hum Reprod (2015) 30:2871–80. doi: 10.1093/humrep/dev227 26373788

[48] Djakow J Svobodova T Hrach K Uhlik J Cinek O Pohunek P . Effectiveness of sequencing selected exons of DNAH5 and DNAI1 in diagnosis of primary ciliary dyskinesia. Pediatr Pulmonol (2012) 47:864–75. doi: 10.1002/ppul.22520 22416021

[49] Castleman VH Romio L Chodhari R Hirst RA de Castro SC Parker KA . Mutations in radial spoke head protein genes RSPH9 and RSPH4A cause primary ciliary dyskinesia with central-microtubular-pair abnormalities. Am J Hum Genet (2009) 84:197–209. doi: 10.1016/j.ajhg.2009.01.011 19200523 PMC2668031

[50] Li AH Hanchard NA Azamian M D'Alessandro LCA Coban-Akdemir Z Lopez KN . Genetic architecture of laterality defects revealed by whole exome sequencing. Eur J Hum Genet (2019) 27:563–73. doi: 10.1038/s41431-018-0307-z PMC6460585 30622330

[51] Yu X Ng CP Habacher H Roy S . Foxj1 transcription factors are master regulators of the motile ciliogenic program. Nat Genet (2008) 40:1445–53. doi: 10.1038/ng.263 19011630

[52] Huang N Zhang D Li F Chai P Wang S Teng J . M-phase phosphoprotein 9 regulates ciliogenesis by modulating CP110-CEP97 complex localization at the mother centriole. Nat Commun (2018) 9:4511. doi: 10.1038/s41467-018-06990-9 30375385 PMC6207757

[53] Guan WJ Peng Y Zi XX Tan KS He TT Zhong NS . Motile ciliary disorders in chronic airway inflammatory diseases: Critical target for interventions. Curr Allergy Asthma Rep (2018) 18:48. doi: 10.1007/s11882-018-0802-x 30046922

[54] Shoemark A Frost E Dixon M Ollosson S Kilpin K Patel M . Accuracy of immunofluorescence in the diagnosis of primary ciliary dyskinesia. Am J Respir Crit Care Med (2017) 196:94–101. doi: 10.1164/rccm.201607-1351OC 28199173 PMC5519960

[55] Lucas JS Barbato A Collins SA Goutaki M Behan L Caudri D . European Respiratory society guidelines for the diagnosis of primary ciliary dyskinesia. Eur Respir J (2017) 49. doi: 10.1183/13993003.01090-2016 PMC6054534 27836958

[56] Li YY Li CW Chao SS Yu FG Yu XM Liu J . Impairment of cilia architecture and ciliogenesis in hyperplastic nasal epithelium from nasal polyps. J Allergy Clin Immunol (2014) 134:1282–92. doi: 10.1016/j.jaci.2014.07.038 25201258

[57] Kennel C Gould EA Larson ED Salcedo E Vickery T Restrepo D . Differential expression of mucins in murine olfactory versus respiratory epithelium. Chem SENSES (2019) 44:511–21. doi: 10.1093/chemse/bjz046 PMC7357245 31300812

[58] Hu K Tan C Lin K Cheng Y Huang H . Effect of endoscopic sinus surgery on irradiation-induced rhinosinusitis in patients with nasopharyngeal carcinoma. Otolaryngology–Head Neck Surg (2008) 139:575–9. doi: 10.1016/j.otohns.2008.07.006 18922347

[59] Zuo W Zhang T Wu DZ Guan SP Liew AA Yamamoto Y . p63(+)Krt5(+) distal airway stem cells are essential for lung regeneration. NATURE (2015) 517:616–20. doi: 10.1038/nature13903 PMC7095488 25383540

[60] Stoddard TJ Varadarajan VV Dziegielewski PT Boyce BJ Justice JM . Detection of microbiota in post radiation sinusitis. Ann Otol Rhinol Laryngol (2019) 128:1116–21. doi: 10.1177/0003489419862583 31304771

[61] Carvalho HA Villar RC . Radiotherapy and immune response: the systemic effects of a local treatment. Clinics (Sao Paulo) (2018) 73:e557s. doi: 10.6061/clinics/2018/e557s 30540123 PMC6257057

[62] De Martino M Daviaud C Vanpouille-Box C . Radiotherapy: An immune response modifier for immuno-oncology. Semin Immunol (2021) 52:101474. doi: 10.1016/j.smim.2021.101474 33741223

[63] Akdis CA . The epithelial barrier hypothesis proposes a comprehensive understanding of the origins of allergic and other chronic noncommunicable diseases. J Allergy Clin Immunol (2022) 149:41–4. doi: 10.1016/j.jaci.2021.11.010 34822880

[64] Akdis CA . Does the epithelial barrier hypothesis explain the increase in allergy, autoimmunity and other chronic conditions? Nat Rev Immunol (2021) 21:739–51. doi: 10.1038/s41577-021-00538-7 33846604

